# Indoor Exposure and Regional Inhaled Deposited Dose Rate during Smoking and Incense Stick Burning—The Jordanian Case as an Example for Eastern Mediterranean Conditions

**DOI:** 10.3390/ijerph20010587

**Published:** 2022-12-29

**Authors:** Tareq Hussein

**Affiliations:** 1 Environmental and Atmospheric Research Laboratory (EARL), Department of Physics, School of Science, The University of Jordan, Amman 11942, Jordan; tareq.hussein@helsinki.fi; 2 Institute for Atmospheric and Earth System Research (INAR/Physics), University of Helsinki, FI-00014 Helsinki, Finland

**Keywords:** fine particles, particle losses, particle number size distribution

## Abstract

Tobacco smoking and incense burning are commonly used in Jordanian microenvironments. While smoking in Jordan is prohibited inside closed spaces, incense burning remains uncontrolled. In this study, particle size distributions (diameter 0.01–25 µm) were measured and inhaled deposited dose rates were calculated during typical smoking and incense stick-burning scenarios inside a closed room, and the exposure was summarized in terms of number and mass concentrations of submicron (*PN_Sub_*) and fine particles (*PM_2.5_*). During cigarette smoking and incense stick-burning scenarios, the particle number concentrations exceeded 3 × 10^5^ cm^−3^. They exceeded 5 × 10^5^ cm^−3^ during shisha smoking. The emission rates were 1.9 × 10^10^, 6.8 × 10^10^, and 1.7 × 10^10^ particles/s, respectively, for incense, cigarettes, and shisha. That corresponded to about 7, 80, and 120 µg/s, respectively. Males received higher dose rates than females, with about 75% and 55% in the pulmonary/alveolar during walking and standing, respectively. The total dose rates were in the order of 10^12^–10^13^ #/h (10^3^–10^4^ µg/h), respectively, for *PN_Sub_* and *PM_2.5_*. The above reported concentrations, emissions rates, and dose rates are considered seriously high, recalling the fact that aerosols emitted during such scenarios consist of a vast range of toxicant compounds.

## 1. Introduction

Incense-burning and aroma products are not widely prohibited worldwide, as some countries still use them extensively [1,2,3,4,5,6,7,8]. In principle, smoking is prohibited in some countries, but this is still not fully obeyed in a large portion of the globe, especially in the Middle East and North Africa (MENA) region [9,10,11,12,13,14,15,16,17,18,19,20,21,22,23], where tobacco smoking (mainly cigarettes and shisha, which is also known as waterpipe, hookah, narghile, or narghila) is still experienced in homes, restaurants, vehicles, malls, government buildings, and offices; shisha smoking is still widely allowed in coffee shops and offered to teenagers [22,24,25,26,27,28,29,30,31,32,33,34,35,36,37,38,39,40,41]. Alternatively, other forms of smoking (e.g., IQOS, e-cigarettes, vaping, etc.) have been introduced as safe or less harmful than tobacco or shisha smoking, but these forms of smoking have not been extensively researched for their possible harmful health effects [42,43,44,45,46]. Nevertheless, some research results have confirmed that these types of alternative smoking types do have harmful health effects [43,47,48,49,50,51,52,53,54,55,56,57,58,59].

Smoking is not only considered harmful for the smoker themselves, but also harmful for the persons nearby, as passive smokers are exposed to the smoke itself being released and transported in the atmosphere, which is known as being a second-hand smoker [47,48,49,50,60,61,62,63,64,65,66,67]. Researchers have also introduced the term “third-hand smokers” [68,69,70,71,72,73,74,75,76,77,78,79], which is not a new issue in public health but is a less well-understood type of smoke exposure. This happens as residual smoke sorbed onto surfaces (furniture, building materials, clothes, etc.) is re-emitted into the atmosphere even after active smoking is ceased. Third-hand smoking exposure pathways can be thought of as the inhalation, ingestion, or dermal uptake of pollutants.

Although the Jordanian Ministry of Health has stated that smoking is forbidden in microenvironments (e.g., houses, schools, offices, etc.), people are still violating this law. In fact, Jordan acquires the highest rate of smoking; 8 out of 10 men consume nicotine products such as cigarettes, shisha (waterpipe, hookah, narghile), and e-cigarettes. According to previous statistics [10,11], about 20% of 13-year-old boys (7.5% girls) in Irbid, Jordan consume nicotine products such as shisha and cigarettes compared to about 11.3% in girls. Contrary to smoking, incense burning does not have any regulations in Jordan. These two types of indoor activities impose serious health issues indoors. It is, therefore, very important to quantify the exposure to air pollution produced during smoking and incense burning for Jordanian conditions, which can be a good representative of several nations in the Eastern Mediterranean region.

Tobacco smoke consists of up of 4000 chemicals, including (varying greatly between the type of smoking) volatile organic compounds (VOCs: Acetonitrile, Acrylnitrile, Benzene, 2,5-Dimethylfuran, 3-Ethenylpyridine, Nicotine, Aldehydes (e.g., Formaldehyde, Acetaldehyde, Acrolein, Propionaldehyde, Methacrolein, etc.), Acetone, Pyrrole, Pyrroline, Pyrrolidine, Phenolics, etc.), polycyclic aromatic hydrocarbons (PAHs: Naphthalene, Acenaphtylene, Acenaphthene, Fluorene, Phenanthrene, Anthracene, Fluoranthene, Pyrene, Benz(a)anthracene, Chrysene, benzo(b)-fluoranthene, benzo(k)-fluoranthene, Benzo(a)pyrene, Indeno(1,2,3-cd)pyrene, Dibenzo(a,h)anthracene, Benzo(g,h,i)perylene), CO, and heavy metals; these are those ones that have been identified as risk factors for health [32,62,80,81,82,83,84,85,86,87,88,89,90,91,92,93,94,95,96,97,98,99,100,101]. Incense burning produces a mixture of particulate matter and a vast range of gases, which includes—and is not limited to—CO, CO_2_, NO_x_, SO_2_, PAHs, VOCs (including benzene, toluene, terpinols, and xylenes), formaldehyde (HCHO), BTEX, and heavy metals [102,103,104,105,106,107,108,109,110,111,112,113,114]. As a matter of fact, air pollution produced during tobacco smoking and incense burning is one of the risk factors for cardiovascular diseases, cancer, respiratory illness, and a wide range of other harmful health outcomes [86,87,93,101,115,116,117,118,119,120,121,122,123,124,125,126,127,128,129,130,131,132]. The World Health Organization (WHO) estimates the mortality rate associated with tobacco smoking as six million people per year, with a projected increase to eight million by the year 2030. Moreover, smoking accounts for six out of eight leading causes of death worldwide and is associated with a large percentage of healthcare costs.

Here, we aim to present the exposure levels and regional inhaled deposited rate in respiratory tracts during the most common combustion activities in Jordanian microenvironments. The investigation included measurements of particle number size distributions (diameter 0.01–25 µm) during typical smoking and incense stick-burning scenarios inside a closed room. The exposure and dose rates were presented in terms of two metrics (number and mass) of submicron and fine particles. In order to put an insight into the exposure scenarios, the regional inhaled deposited dose rates were calculated for an adult male or female during two of the main human activities (walking and standing).

## 2. Materials and Methods

### 2.1. Measurement Setup

The measurement setup was according to the description provided by Hussein et al. [133]. The setup consisted of the following portable instruments: (1) condensation particle counters (CPC, 3007-2, TSI, Shoreview, MN, USA), (2) handheld optical counter (P-Trak 8525, TSI, Shoreview, MN, USA), and (3) and a handheld optical particle counter (AeroTrak 9306-V2, TSI, Shoreview, MN, USA).

The CPC and P-Trak measure submicron particle number concentrations with cutoff sizes of 10 nm and 25 nm, respectively. The sampling flow rate in these two instruments was 0.1 lpm (inlet flow rate 0.7 lpm). The maximum measurable concentrations were ~10^5^ cm^−3^ and 5 × 10^5^ cm^−3^, respectively. These instruments were operated at 1 s time resolution. Using the AeroTrak extended measured particle diameters to reach 25 µm and allowed for 6 particle size channels: 0.3, 0.5, 1, 2.5, 10, and 25 µm (optical diameter). The AeroTrak sampling time resolution was 30 s at a flow rate of 2.83 lpm.

### 2.2. Exposure Scenarios

The exposure scenarios included smoking (cigarette and shisha) and incense stick burning. The scenarios were performed inside an office room (5 × 2.7 × 2.7 m^3^), which was fully furnished and had its window and door closed. Each scenario was repeated four times. The room was naturally ventilated.

The cigarette smoking was made by burning two cigarettes within a short time (<3 min). Two shishas were smoked at the same time for a period between 30 min and 90 min. The incense stick scenario included burning two sticks (~20 cm long) at the same time for five minutes.

After finishing the smoking and the incense stick burning, the room was kept closed and unoccupied until the particle number concentration reached a level close to what was before the scenario was started. This took a period of longer than three hours.

### 2.3. Data Handling

The measured particle number size distribution (eight channels within 0.01–25 µm) was generated by processing the data by the three instruments listed in the previous section [22,133]. Basically, the use of three instruments with different cutoffs has the advantage of generating the particle number size distribution by subtracting the concentrations measured with the different instruments. The most important part here is to harmonize the aerosol database measured with different instruments.

The harmonized aerosol database was processed for 1 min average. The particle number size distribution (10 nm–25 µm, 8 channels) was generated by calculating the concentration differences between different instruments: 10–25 nm (CPC and P-Trak difference), 25–300 nm (P-Trak and Aerotrak difference), and 6 channels in the AeroTrak (0.3–0.5 µm, 0.5–1 µm, 1–2.5 µm, 2.5–5 µm, 5–10 µm, and 10–25 µm).

The size-fractionated number and mass concentrations were calculated over the specified particle size range:

(1) $$PN_{D_{p1}-D_{p2}}=\overset{D_{p2}}{\underset{D_{p1}}{\int }}n_{N}^{0}\cdot dlog_{10}\left(D_{p}\right)$$

(2) $$PM_{D_{p1}-D_{p2}}=\overset{D_{p2}}{\underset{D_{p1}}{\int }}n_{M}^{0}\cdot dlog_{10}\left(D_{p}\right)$$

where $n_{N}^{0}=dN/d\log_{10}\left(Dp\right)$ is the lognormal particle number size distribution and $n_{M}^{0}=dM/d\log_{10}\left(Dp\right)$ is the lognormal particle mass size distribution. Here, the particles were assumed spherical, with unit density (*ρ_p_* = 1000 kg/m^3^).

### 2.4. Particle Emission Rate and Loss Rate Calculation—A Simple Indoor Aerosol Model

The size-specific emission rate and losses of aerosol particles were calculated by utilizing a simple indoor aerosol model [23,134,135,136]. The basic principle is based on the change rate of particle number concentrations calculated for each particle size. At first, the particle loss rate was calculated. Then, the emission rate was calculated and corrected for the particle losses.

While in general, indoor aerosol origin is a combination of indoor or outdoor sources, the indoor aerosol sources in this study are dominated by those sources that are due to smoking and incense burning. The indoor aerosols are eventually lost from the atmosphere by either dry deposition or removal via air exchange. Complex dynamic processes (coagulation, condensation, chemical reactions, etc.) were ignored. Accordingly, indoor aerosols concentrations can be described by the mass balance equation:

(3) $$\frac{dI_{i}}{dt}=P_{i}\lambda O_{i}-\left(\lambda +\lambda_{d,i}\right)I_{i}+S_{in,i}$$

where *t* is the time; *I* and *O* are the indoor and outdoor concentrations of the aerosol particles, respectively; *P* is the penetration factor of aerosol particles while being transported from the outdoor air into the indoor air; *λ* is the ventilation rate; *λ_d_* is the dry deposition rate of aerosol particles onto available indoor surfaces; and *S_in_* represents the emission rates from an indoor source. The subscript *i* represents a certain particle size range. A well-mixed indoor air domain was ensured because the room size was small enough and a fan was used to produce air convection.

In Equation (3), the second and third terms on the right-hand side are the particle losses (dry deposition and removal by ventilation) and emission rate from an indoor source, respectively. Right after an indoor source is terminated, Equation (3) can be rewritten to calculate the particle losses as

(4) $$\frac{dI_{i}}{dt}\to -\left(\lambda +\lambda_{d,i}\right)I_{i}$$

and right after a source is started, it can be rewritten to calculate the emission rate as

(5) $$\frac{dI_{i}}{dt}+{\left\{\left(\lambda +\lambda_{d,i}\right)I_{i}\right\}}_{emperical}\to S_{in,i}$$

Here, Equation (4) is built on the fact that indoor aerosols of indoor origin are dominant (i.e., *PλO* << (*λ* + *λ_d_*)*I*). In Equation (5), the source term is assumed to be dominant (i.e., *S_in_* << *PλO* − (*λ* + *λ_d_*)*I*) but it needs to be corrected for the particle losses.

### 2.5. Reigional Inhaled Deposited Dose in the Respiratory Tracts

**Figure 1:**
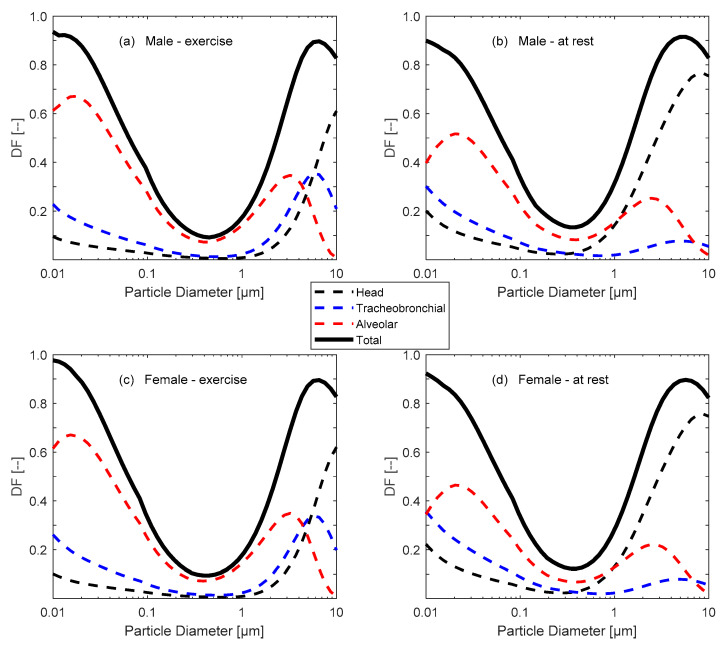
Deposition fraction (*DF*) curves in an adult: (**a**) male exercising, (**b**) male at rest, (**c**) female exercising, and (**d**) female at rest.

The measured particle number size distribution can be used to calculate the deposited fraction of aerosols in the respiratory tracts for a specific particle diameter range (*D_p1_*–*D_p2_*). Here, we followed our previous approach described elsewhere [137,138,139,140]. Here, the respiratory tracts were divided into three main regions: head/throat (H), tracheobronchial (TB), and pulmonary/alveolar (P/Alv), according to the ICRP and MPPD models [141,142,143], and the calculations require the following:

- The predefined particle deposition fraction (*DF*(*D_p_*)) in the respiratory tracts during exercise or rest (Figure 1) [141,142,143];
- The minute ventilation (*V_E_*, m^3^/h), which is the volume of breathed air per time (Table 1);
- The measured particle number size distribution ( $n_{N}^{0}\left(D_{P}\right)$, particles/cm^3^):

(6) $$\mathrm{Dose}\;\mathrm{Rate}=\int_{D_{P}1}^{D_{P}2}V_{E}\;DF\;n_{N}^{0}\;f\cdot dlog\left(D_{P}\right)$$

Here, *f* is a metric conversion for the particle concentration: it is 1 for particle number, and for particle mass it is *ρ*_p_*D_p_*^3^π/6.

**Table 1:** Minute ventilation (*V_E_*, m^3^/h) for adult subjects.

Activity	Female	Male	*DF* Curve Type
Walking (4.0 km/h)	1.20	1.38	Exercise
Standing	0.48	0.66	at rest

## 3. Results and Discussion

### 3.1. Temporal Variation, Size Distirbutions, and Particle Losses

**Figure 2:**
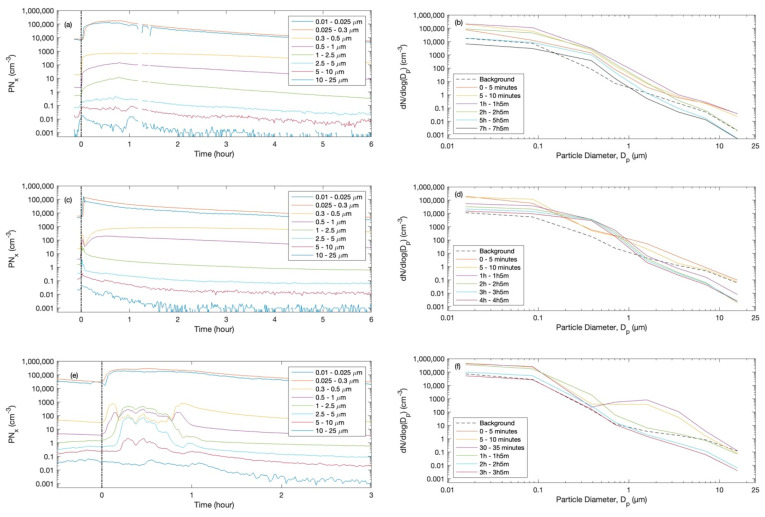
Particle number concentrations and size distributions during (**a**,**b**) incense stick burning, (**c**,**d**) cigarette smoking, and (**e**,**f**) shisha smoking. The left panel is the time series of different particle size fraction concentrations, where the vertical dashed line at zero hour indicates the start of the scenario, and the right panel is selected particle number size distributions during different time periods.

As examples, the total particle number concentrations during one case of each exposure scenario (cigarette, shisha, and incense) are presented in Figure 2 (left panel). When the scenario started, the fine particle concentrations increased by several factors. After each scenario was over (i.e., smoking or burning), the concentrations decayed gradually as a result of the indoor–outdoor air exchange and dry deposition. During the cigarette and incense scenarios, the concentrations increased continuously until the end of smoking or burning, but during shisha smoking, the concentrations varied as a result of the puffing sequence or the addition of charcoal. The pollution emitted during smoking depends on the puffing and smoking behaviour [144,145,146]. The emission factor is also dependent on the shisha setup (bowel size, head size, hose length, etc.) [147]. In fact, the temporal variation of the particle concentrations agreed well with previous studies that reported smoking (cigarette and shisha) and incense burning [106,148,149,150].

**Figure 3:**
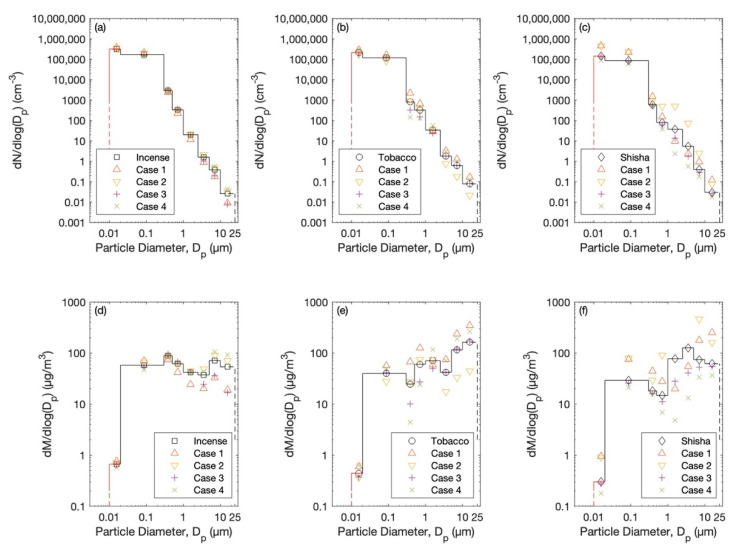
Mean particle number size distributions during each case of the different scenarios: (**a**) incense stick burning, (**b**) cigarette smoking, and (**c**) shisha smoking. (**d**–**f**) The corresponding mean particle mass size distributions.

**Figure 4:**
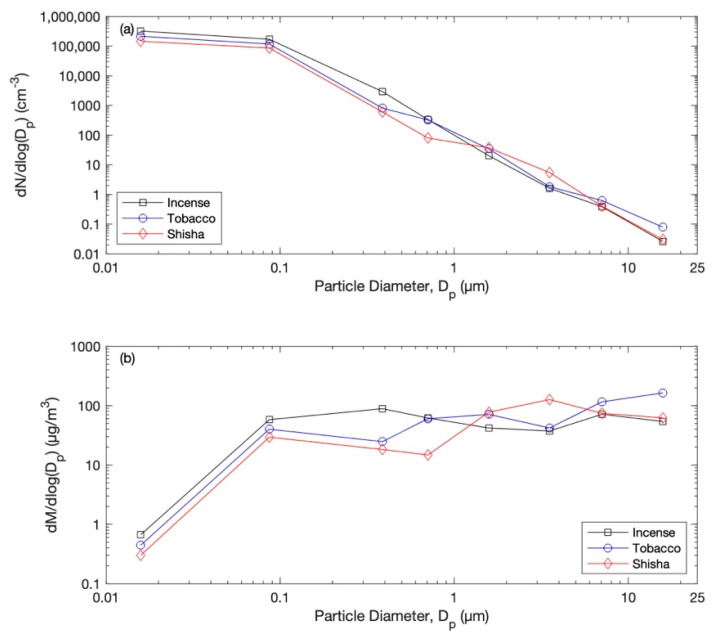
Overall mean (**a**) particle number size distributions and (**b**) corresponding mass size distributions by assuming spherical particles and unit density.

**Figure 5:**
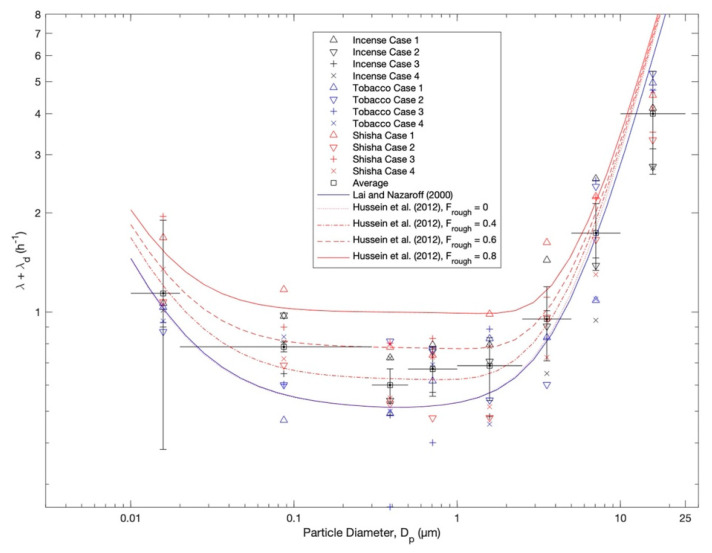
Total particle losses of aerosol particles calculated for each scenario compared to the three-layer model calculations by Lai and Nazaroff [151] for smooth surfaces and Hussein et al. [152] for rough surfaces. The model calculation was made for a room (5 × 2.7 × 2.7 m^3^), friction velocity u* = 10 cm/s, and ventilation rate *λ* = 0.5 h^−1^. The surface roughness parameter (*F_rough_*) is zero for a smooth surface.

Selected particle number size distributions during one case of each exposure scenario are presented in Figure 2 (right panel). The mean particle number and mass size distributions during the almost-steady-state concentration level are shown in Figure 3 and Figure 4. The total particle losses—which are dependent on many factors, including: the particle size distribution, indoor–outdoor air exchange, and dry deposition for each case of the scenarios—are presented in Figure 5. The average total particles losses were 0.45–2 h^−1^ for particles smaller than 0.1 µm. Those for micron particles were 0.45–5.5 h^−1^. According to the losses of particles within the range 0.1–1 µm, the ventilation rate (*λ*) can be around 0.5 h^−1^. This value agrees with typical values of natural ventilation.

The three-layer deposition model calculation, as described by Lai and Nazaroff [151], shows underestimation for the total particle losses; room volume 5 × 2.7 × 2.7 m^3^ and friction velocity u* = 10 cm/s. This underestimation is expected because the Lai and Nazaroff calculation [151] is for smooth surfaces. The roughness of indoor surfaces can be taken into account by the three-layer deposition model described by Hussein et al. [152]; the surface roughness parameter (*F_rough_*) can be smaller than 0.8.

The incense smoke (inside a small stainless-steel test chamber, 1.2 × 1.2 × 1.2 m^3^) was characterized by a skewed unimodal particle number size distribution to small diameters and varying peak values of 75–170 nm (mean 130 nm) and total particle losses of 1.% 2.32 h^−1^ (mean 2.4 h^−1^) [150]. In the same study, the cigarette smoke was characterized by a unimodal particle number size distribution, a peak value at around 110 nm, and mean total particle losses of 3.9 h^−1^ [150]. In another study inside a small chamber (1.016 × 0.660 × 1.600 m^3^), the geometric mean diameter of the incense smoke particles was nearly unimodal, with a geometric mean of 80–15 nm [153]. The PM_2.5_ of incense smoke inside a church showed a loss rate in the range of 1.1 × 10^−2^–5.5 × 10^−2^ min^−1^ (mean 3.2 × 10^−2^ min^−1^) [8].

### 3.2. Exposure

#### 3.2.1. Incense Sticks

During the incense scenario and right after the two sticks started burning, the fine particle concentrations increased quickly, especially those in the ultrafine particle size range (i.e., UFP, diameter < 0.1 µm). The total particle number concentrations were higher than 3 × 10^5^ cm^−3^ (corresponding to >110 µg/m^3^) (Figure 2a). The duration of the high-concentration period was as long as the two sticks were burning. It is noticed from Figure 2a that the micron particle concentrations increased before starting the incense stick burning; this was basically due to the fact that the room was opened to enter and start the scenario. The evolution of the particle number size distributions is presented in Figure 2b, which clearly shows how the burning affected the fine particles.

In a previous investigation conducted inside a room (2.53 × 2.50 × 5.15 m^3^) [106], the total particle number concentration was reported as 9.1 × 10^4^ cm^−3^ during incense burning. In another study conducted inside a small chamber (1.016 × 0.660 × 1.600 m^3^), the incense smoke (four incense sticks were burned at each of the four corners of the chamber) showed a highest concentration of 1.1 × 10^6^–2.4 × 10^6^ cm^−3^ [153]. The total particle number concentrations observed inside an empty room with dimensions of 2.7 × 3.8 × 3.1 m^3^ for two types of incense varied within 1.6 × 10^5^–2.9 × 10^5^ cm^−3^ (mean 2.2 × 10^5^ cm^−3^) [108]. The UFP concentrations (inside a stainless-steel test chamber 1.2 × 1.2 × 1.2 m^3^) [150] had a maximum concentration of the incense smoke within the range of 5.7 × 10^4^–6.1 × 10^5^ cm^−3^ (mean 2.9 × 10^5^ cm^−3^). In the European Accredited (EA) Laboratory of Industrial Measurements (LAMI) at the University of Cassino and Southern Lazio, a test of three incense-burning scenarios yielded 4.1 × 10^6^–9.8 × 10^6^ cm^−3^ (PM_2.5_ 6.3–14.6 mg/m^3^) [105].

As a cultural and religious custom, incense burning is widely used in some countries [3,4,5,6,7,8]. The PM_2.5_ concentrations inside a church reached values of 36–123 μg/m^3^ (mean 56 μg/m^3^), equivalent to 5 × 10^3^–1.4 × 10^4^ cm^−3^ (mean 6.3 × 10^3^ cm^−3^) [8]. The average PM_2.5_ concentration at ten temples was reported to be 660 µg/m^3^, and at two crematoriums, it was reported to be 1050 µg/m^3^, as a result of incense burning [7]; these values were ten and five times higher than the outdoor concentration reported at the nearby ambient air-monitoring station.

#### 3.2.2. Cigarette

During cigarette-smoking scenarios (Figure 2c,d), the total particle number concentrations were higher than 3 × 10^5^ cm^−3^. The duration of smoking two cigarettes was less than three minutes, and consequently, the aerosol emission span time was very short. Initially, fine particles of all sizes were affected as a result of opening the office to start the cigarette-smoking scenario. However, it was evident that particles smaller than 0.3 µm were the most affected, because their concentrations immediately increased significantly right after starting the scenario. Interestingly, after 10 min of starting the smoking, when the UFP concentrations started to decrease, the concentrations of the accumulation mode (diameter 0.1–1 µm) started to increase as a result of coagulation of the emitted smoke particles. This was also evident in the evolution of the particle number size distributions shown in Figure 2d. The lifetime of the emitted smoke particles were longer than those emitted from the incense burning.

The UFP concentrations of cigarette smouldering evaluated inside in a stainless-steel test chamber (1.2 × 1.2 × 1.2 m^3^) were as high as 1.2 × 10^6^ cm^−3^ [150]. The PM_2.5_ exposure levels as second-hand smoke (five types of Vogue cigarettes) ranged between 1.3 and 2.2 mg/m^3^ [154]. In another test inside a small chamber (2.88 m^3^), the PM_2.5_ exposure levels as second-hand smoke were 0.7–1.5 mg/m^3^ during the testing of five types of Marlboro cigarette [155].

Particle concentrations in the mainstream are extremely higher than those inhaled by a passive smoker in typical indoor, outdoor, and occupational environments, as well as vehicles: typically in the range of 10^3^–10^6^ cm^−3^ [156,157,158,159,160,161,162,163,164,165,166,167,168,169,170,171,172,173,174,175,176,177,178,179]. It was reported that in the mainstream, the particle number concentration may reach values in the order of 10^8^ cm^−3^, as tested for five different brands (B&H, Camel, Marlboro, Merit, Wenston) and characterized by a single lognormal mode with a geometric mean diameter of 180 nm and 220 nm [180], which agreed well with this study, as well as others reported in the literature [164,181]. In another study, it was shown to be in the range of 10^5^–10^9^ cm^−3^ in the mainstream [182].

#### 3.2.3. Shisha

The scenario of shisha smoking took about an hour, during which the particle number concentrations of all particle sizes smaller than 10 µm were significantly high (total > 5 × 10^5^ cm^−3^) when compared to the background concentrations (Figure 2e). Interestingly, the UFP concentrations were steadily high throughout the scenario, but the concentrations of other particle size fractions within the range of 0.1–10 µm were continuously variable. During shisha smoking, the smokers were frequently moving, and consequently, dust resuspension occurred, as clearly seen in the particle number size distributions shown in Figure 2f. The lifetime of the emitted aerosols from shisha smoking (less than 2 h) was shorter than those emitted from cigarette smoking or incense burning.

The literature about shisha smoke characterization is fairly limited when compared to cigarette smoke or incense burning. However, and as pointed out before, shisha smoke temporal variation in this study was similar to what was previously reported in other studies, and can be explained by puffing intensity and frequency, as well as the addition/changing of charcoal [148,149]. In our previous study reported for indoor air quality inside Jordanian dwellings, houses with smoking activities had aerosol concentrations (both number and mass) higher than houses without smoking activities [22]. Compared to this study, which was inside an office (~36.5 m^3^), these houses reflected real-life conditions inside relatively large indoor environments (apartments and houses), revealing that the submicron particle number concentrations were as high as 1.5 × 10^5^ cm^−3^ (equivalent to PM_2.5_ ~100 µg/m^3^) inside the dwellings with cigarette smoking. Those with shisha smoking experienced even higher concentrations ~4 × 10^5^ cm^−3^ (equivalent to PM_2.5_ ~130 µg/m^3^).

As expected and reported previously [148], cigarette smoke particles were generally larger in size than shisha smoke; the count mean diameter (CMD) of the cigarette smoke particles was 130 nm (σ_g_ = 2.0) whereas the size distribution of the particles emitted from shisha was bimodal (one peak below 10 nm and another peak at 65 nm). The mean particle number concentration in the aerosol chamber over the entire measurement period was 1.5 × 10^5^ and 1.9 × 10^5^ cm^−3^ for the cigarette and waterpipe smoke, respectively.

In a similar study, a shisha-smoking session was carried out in a room (floor area 20 m^2^, 4 h smoking), resulting a median particle number concentration of 2.9 × 10^5^ cm^−3^ (95% values 5.5 × 10^5^ cm^−3^), which was equivalent to a PM_2.5_ of about 390 µg/m^3^ (95% value 740 µg/m^3^) [16].

### 3.3. Particle Emission Rates

**Figure 6:**
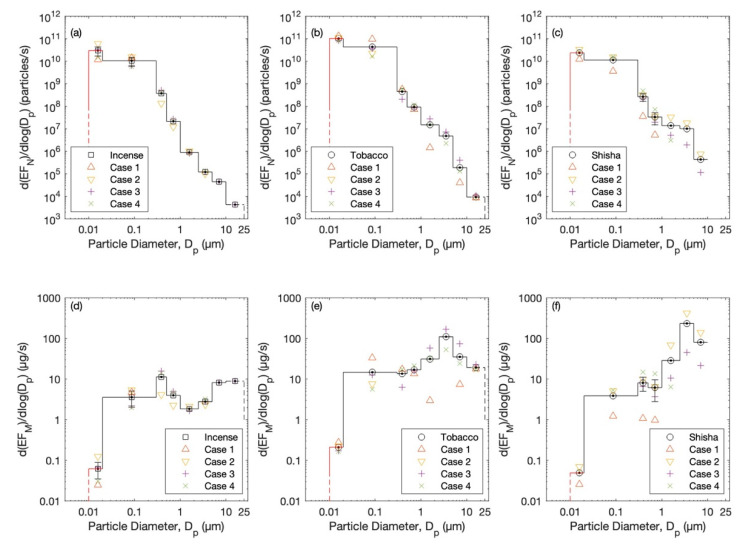
Mean emission rates in the form of particle number size distributions during each case of the different scenarios: (**a**) incense stick burning, (**b**) cigarette smoking, and (**c**) shisha smoking. (**d**–**f**) The corresponding mean particle mass size distributions.

The size-specific particle emission rate is shown in Figure 6. On average, the total number of particles emitted while burning two incense sticks at the same time was about 1.9 × 10^10^ particles/s; this is equivalent to about 7 µg/s. As for smoking, the total particles emitted were about 6.8 × 10^10^ and 1.7 × 10^10^ particles/s, respectively, for cigarettes and shisha. Although the total number emitted during cigarette smoking was more than that during shisha smoking, the total particle mass emitted during shisha smoking (about 120 µg/s) was more than that during cigarette smoking (about 80 µg/s).

The literature has limited and few studies reporting the emission factors during incense-burning and cigarette-smoking scenarios. To our knowledge, emission factors were not reported before for shisha. Inside a small chamber (1.016 × 0.660 × 1.600 m^3^) used to characterize the physical properties of particles in incense smoke (four incense sticks were burned at each of the four corners of the chamber) the emission rate was 5.1 × 10^12^–1.42 × 10^13^ particles/h [153]. In the European Accredited (EA) Laboratory of Industrial Measurements (LAMI, University of Cassino) the emission rate was 4.5 × 10^14^–1.1 × 10^15^ particles/h (PM_2.5_ 19.4–50.3 mg/h) during three incense-burning scenarios [105]. It was previously reported for 23 different types of incense (sticks, cones, powder, smudge bundle, coil, rope, and rocks) that the emissions of particulate matter were with a PM_2.5_ emission rate of 7–202 mg/h [183]. The UFP emission rate of incense burning carried out in a 1.2 × 1.2 × 1.2 m^3^ stainless-steel test chamber was 0.4 × 10^11^ particles/min [150]. The PM_2.5_ emission rates were 1.1 × 10^2^–3.9 × 10^2^ particles/cm^3^min (mean 2.5 × 10^2^ particles/cm^3^min), equivalent to 1.4–3.9 μg/m^3^min (mean 56 μg/m^3^min) [8].

The UFP emission rate of cigarette smouldering carried out in a 1.2 × 1.2 × 1.2 m^3^ stainless-steel test chamber was 3.4 × 10^11^ particles/min [150], with a unimodal particle number size distribution and peak value at around 110 nm. The temporal variation was similar to the one observed in this study, with a maximum concentration of 1.2 × 10^6^ cm^−3^ and mean total particle losses of 3.9 h^−1^. The PM_2.5_ emission rate of 1.2–2 mg/m^3^s was derived from the exposure levels as second-hand smoke (five types of Vogue cigarette) [154]. In another test made inside a small chamber (2.88 m^3^), the PM_2.5_ emission rate was 580–1250 mg/m^3^s while testing five types of Marlboro cigarette [155].

### 3.4. Regional Inhaled Deposited Dose

In order to put an insight into the exposure scenarios, the regional inhaled deposited dose rates were calculated for an adult male or female (Table 2 and Table 3) during two of the main human activities (walking 4 km/h and standing) and being exposed to three scenarios: incense burning, cigarette smoking, and shisha smoking. The metrics were the number concentration of submicron particles (*PN_Sub_*, diameter < 1 µm) and particulate matter (*PM_2.5_*).

In general, and regardless of the type of combustion scenario and concentration metric, the total inhaled dose rate was higher in males than females. This is basically due to the fact that male minute ventilation (*V_E_*) is higher, and the deposition fraction (*DF*) curves are slightly different for males than females. On average, the regional inhaled dose rates during the walking scenario were about 7%, 18%, and 75%, respectively, for the head and throat airways (H), tracheobronchial (TB), and pulmonary/alveolar (Alv) regions. During standing, the corresponding dose rates were 25%, 20%, and 55%, respectively. The differences in the regional dose rates reflect the changes in the *DF* curves between resting and exercising.

During standing, and with respect to the combustion scenario, the total dose rate of submicron particles (*PN_Sub_*, Table 2) was the highest during incense burning (6.3 × 10^12^ #/h for males versus 4.6 × 10^12^ #/h for females) and the lowest during cigarette smoking (1.1 × 10^12^ #/h for males versus 0.8 × 10^12^ #/h for females). Considering the total dose rate of fine particles (*PM_2.5_*, Table 3) during standing, the highest was during shisha smoking, with 3.8 mg/h for males (versus 2.5 mg/h for females), and the lowest was 0.3 mg/h for males (versus 0.2 mg/h for females).

Similarly, the total dose rate during walking (4 km/h) was the highest during incense burning (13.7 × 10^12^ #/h for males versus 12 × 10^12^ #/h for females) and the lowest was during cigarette smoking (2.3 × 10^12^ #/h for males versus 2 × 10^12^ #/h for females). With respect to *PM_2.5_*, the total dose rate was the highest during shisha smoking, with 5.3 mg/h for males (versus 4.6 mg/h for females), and the lowest was 0.5 mg/h for both males and females.

It is interesting to mention here that the higher dose rates calculated during walking when compared to standings are attributed to the fact that the minute ventilation (*V_E_*) is higher during exercise (i.e., walking) than during resting (i.e., standing). In addition, the *DF* curves are slightly different between the two cases. However, the main effect comes from the minute ventilation (*V_E_*).

The calculated dose rates can be converted to actual cumulative dose in the respiratory tracts by multiplying the dose rate by the exposure time (hours). Without performing the calculations, the cumulative dose is considerably high that it can impose serious health risk, especially knowing that the chemical combustion of the smoke consists of high contents of toxic compounds and heavy metals.

## 4. Conclusions

Tobacco smoking and incense burning are commonly practiced in Jordanian microenvironments. While smoking in Jordan is prohibited inside closed spaces, incense burning remains uncontrolled.

In this study, the particle size distributions (diameter 0.01–25 µm) were measured inside a room replicating real-life conditions in a Jordanian dwelling. The measurement was made during typical smoking and incense stick-burning scenarios as examples of common indoor aerosol sources and exposure. The results were summarized in terms of number and mass concentrations of submicron and fine particles. The particle losses and emission rates were calculated by utilizing a simple indoor aerosols model.

During cigarette smoking and incense stick-burning scenarios, the particle number concentrations exceeded 3 × 10^5^ cm^−3^. During shisha smoking, they exceeded 5 × 10^5^ cm^−3^. The average emission rates were estimated at 1.9 × 10^10^, 6.8 × 10^10^ and 1.7 × 10^10^ particles/s, respectively, for incense, cigarettes, and shisha. That corresponded to about 7, 80, and 120 µg/s, respectively. Interestingly, the exposure levels were almost similar for incense and cigarette-smoking aerosols, but the emission rate from cigarette smoking was about three times that during incense burning. This was expected, because the smoking occurred for a shorter time than the burning of the incense sticks.

In general, and regardless to the type of combustion scenario and concentration metric, males received higher dose rates than females, with average percentiles in the different regions of the respiratory tracts of 7%, 18%, and 75%, respectively, for the head and throat airways (H), tracheobronchial (TB), and pulmonary/alveolar (Alv) regions during walking. During standing, the corresponding dose rates were 25%, 20%, and 55%, respectively. The total dose rates during standing were in the order of 10^12^ #/h and 10^3^ µg/h, respectively, for submicron particle number concentration (*PN_Sub_*) and *PM_2.5_*. During walking (4 km/h), they were in the order of 10^13^ #/h and 10^4^ µg/h, respectively.

The exposure levels, emissions rates, and inhaled deposited dose rates during the studied scenarios of smoking and incense burning are considered seriously high, recalling the fact that aerosols emitted during such scenarios consist of a vast range of polycyclic aromatic hydrocarbons (PAHs), volatile organic compounds (VOCs), and heavy metals, in addition to harmful gases such CO, CO_2_, NO_x_, and SO_2_.

By all means, the Jordanian authorities have to take immediate actions to enforce the prohibition of cigarette smoking indoors and develop regulations for incense stick burning.

## Figures and Tables

**Table 2:** Regional inhaled deposited dose rate (×10^9^ #/h) calculated based on submicron particle number (*PN_Sub_*, diameter < 1 µm) concentration for adult males and females during two main activities (walking 4 km/h and standing). Abbreviations H, TB, and Alv are for head and throat airways, tracheobronchial, and pulmonary/alveolar regions, respectively.

		Male				Female			
		H	TB	Alv	Total	H	TB	Alv	Total
Walking	Incense	1000	2500	10,200	13,700	900	2300	8700	12,000
	Cigarette	200	400	1700	2300	200	400	1500	2000
	Shisha	800	1800	7700	10,300	700	1700	6600	9000
Standing	Incense	900	1500	3800	6300	700	1300	2500	4600
	Cigarette	200	300	600	1100	100	200	400	800
	Shisha	700	1100	2900	4700	600	1000	1900	3400

**Table 3:** Regional inhaled deposited dose rate (µg/h) calculated based on submicron particle mass concentration (*PM_2.5_*) for adult males and females during two main activities (walking 4 km/h and standing). Abbreviations H, TB, and Alv are for head and throat airways, tracheobronchial, and pulmonary/alveolar regions, respectively.

		Male				Female			
		H	TB	Alv	Total	H	TB	Alv	Total
Walking	Incense	190	410	1910	2500	160	370	1600	2130
	Cigarette	40	90	400	520	30	80	340	450
	Shisha	410	930	3920	5270	370	820	3390	4580
Standing	Incense	340	220	820	1380	240	200	520	970
	Cigarette	100	40	170	310	70	40	110	220
	Shisha	1670	350	1750	3760	1150	300	1090	2540

## Data Availability

Data are available upon request.
